# Can *Acanthamoeba* Harbor Monkeypox Virus?

**DOI:** 10.3390/microorganisms11040855

**Published:** 2023-03-27

**Authors:** Ruqaiyyah Siddiqui, Jibran Sualeh Muhammad, Ahmad M. Alharbi, Hasan Alfahemi, Naveed Ahmed Khan

**Affiliations:** 1 College of Arts and Sciences, American University of Sharjah, Sharjah 26666, United Arab Emirates; 2 Department of Medical Biology, Faculty of Medicine, Istinye University, Istanbul 34010, Turkey; 3 College of Medicine, University of Sharjah, Sharjah 27272, United Arab Emirates; 4 Department of Clinical Laboratory Sciences, College of Applied Medical Sciences, Taif University, Taif 21944, Saudi Arabia; 5 Department of Medical Microbiology, Faculty of Medicine, Al-Baha University, Al-Baha 65799, Saudi Arabia

**Keywords:** protozoa, trojan horse, monkeypox virus, *Acanthamoeba*, disease, transmission

## Abstract

*Acanthamoeba* is well known to host a variety of microorganisms such as viruses, bacteria, protozoa, and yeast. Given the recent number of cases of monkeypox infection, we speculate that amoebae may be aiding viral transmission to the susceptible hosts. Although there is no confirmatory evidence to suggest that *Acanthamoeba* is a host to monkeypox (a double-stranded DNA virus), the recent discovery of mimivirus (another double-stranded DNA virus) from *Acanthamoeba*, suggests that amoebae may shelter monkeypox virus. Furthermore, given the possible spread of monkeypox virus from animals to humans during an earlier outbreak, which came about after patients came in contact with prairie dogs, it is likely that animals may also act as mixing vessel between ubiquitously distributed *Acanthamoeba* and monkeypox virus, in addition to the environmental habitat that acts as an interface in complex interactions between diverse microorganisms and the host.

## 1. Introduction

Monkeypox is a rare viral disease affecting humans and animals and is caused by an Orthopoxvirus [1]. The disease is endemic to Africa, with limited cases emerging outside this region, normally linked with travel or through contact with material contaminated with the virus such as imported animals [2]. However, since 7 February 2023, a staggering 85,645 cases have now been reported worldwide, which has alarmed public health officials and scientists due to emergence around the globe [3,4]. This number of cases is significantly greater than the entire number identified outside the continent since 1970, when the virus was first discovered to cause disease in humans. Due to the unprecedented global spread of the disease outside of formerly endemic nations in Africa and the need for international cooperation to fight this previously neglected disease, the World Health Organization (WHO, Geneva, Switzerland) proclaimed monkeypox a public health emergency of international concern in July 2022 [5].

Clinical symptoms of monkeypox infection in humans and animals are comparable to those of smallpox infection [6]. Flu-like illness, fever, and lymphadenopathy are the most typical human monkeypox symptoms seen in endemic locations. Symptoms comprise fever, muscle pains, headache, fatigue, and swollen lymph nodes, followed by a rash [7]. These are followed by the onset of rashes frequently on the face, arms, or legs. Monomorphic lesions, frequently with centrifugal distribution, typically show up on the face in monkeypox [8]. On occasion, lesions could show up on the palms, soles, or genitalia. During transmission of the virus, the incubation period might be anywhere between 3 and 20 days [9].

**Figure 1:**
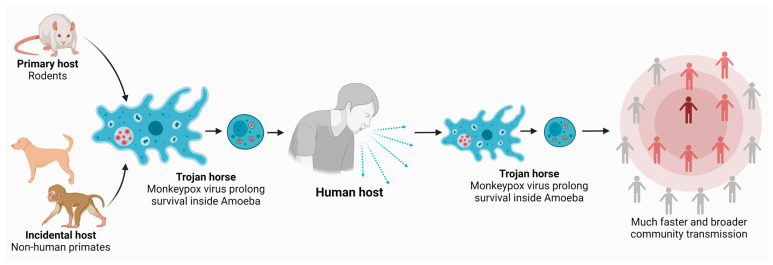
Amoebae may be aiding monkeypox virus in the environmental survival and/or transmission to susceptible hosts. Primary hosts, such as rodents, and incidental hosts, such as monkeys and dogs, shed the monkeypox virus in their bodily secretions. Amoebae in the environment harbor the virus and carry it to the human host. Later, the human host releases more virus into the environment, which is taken up by the amoebae acting as a Trojan horse and facilitating much faster and broader community transmission.

More importantly, these cases have no established travel links to endemic areas; nonetheless, epidemiological investigations are ongoing [3]. It has been suggested that the early spread of the virus may have been linked to two mass events in Spain and Belgium, and cases were predominantly observed among homosexual men [3]. The question that is the subject herein is how does the monkeypox virus (a double-stranded DNA virus) survive in the environment? Notably, Legionnaires’ disease, caused by *Legionella pneumophila,* was due to the presence of bacteria inside air-conditioning units; however, how *L. pneumophila* survived in these air-conditioning units was not known [10]. Further work demonstrated that *L. pneumophila* were able to survive in harsh environments by taking up refuge inside free-living amoebae, i.e., *Acanthamoeba*. More recently, a double-stranded DNA virus, i.e., mimivirus, was discovered in amoebae. Given the ability of *Acanthamoeba* to host a variety of microbes (reviewed in [11]), some of which can cause infections (Figure 1), we speculate that amoebae may shelter monkeypox. This is not a novel concept. It is now well known that “hyperparasitism”, i.e., a parasite inside another parasite, is a survival and transmission mechanism employed by microbes. *Acanthamoeba* is well regarded as one of the most ubiquitous protozoans. It has been isolated from a variety of environments including soil, water, and air. It is also well known to host a variety of microbes including yeast, bacteria, protozoa, and viruses. It was the first amoeba to be described as a host to *Legionella* spp. [10], and the recently discovered mimivirus [12]. Based on its properties, it is suggested as the Trojan horse of the microbial world in the environment (reviewed in [11]). This is strengthened with the fact that *Acanthamoeba* forms airborne cysts that allow it to travel long distance while harboring other microbes. *Acanthamoeba* cysts are highly resistant to physiological, chemical, and radiological conditions [13]. Because of these reasons, it is reasonable to suggest that *Acanthamoeba* may act as a host for monkeypox, and this ought to be explored in future studies and is the subject of this review. However, other free-living amoebae (in addition to *Acanthamoeba*) that can serve as a reservoir for bacteria/viruses should also be considered as potential hosts for monkeypox.

## 2. Monkeypox Epidemiology

In the 10 years following the first human case in 1970 [14,15], there were 59 documented cases of human monkeypox in western and central Africa, with a fatality rate of 17% in children under the age of 10 [5]. The World Health Organization (WHO) kept track of human monkeypox cases after smallpox was eradicated and routine smallpox vaccinations were stopped in 1980 because of the fear that decreased smallpox immunization rates may increase population susceptibility to monkeypox [5]. In the Democratic Republic of the Congo, there were three spatiotemporal clusters indicative of potential monkeypox epidemics from 2000 to 2015, including 760 cases that were confirmed in the lab between 2005 and 2007 [16]. Likewise, after nearly 40 years with no cases reported, an upsurge in monkeypox cases was observed in Nigeria as of 2017 [17].

In various African nations, including Cameroon, Benin, Gabon, Central African Republic, Democratic Republic of the Congo, Ghana (found only in animals), Liberia, Côte d’Ivoire, Nigeria, Sierra Leone, and South Sudan, monkeypox was thought to be endemic [18]. With more than a thousand suspected cases and 58 fatalities between January and May 2022, the Democratic Republic of the Congo was the nation with the highest number of reports [5].

Of concern, in 2022, there was a global resurgence of monkeypox cases, with a high infection incidence in non-endemic nations. According to a recent report [19], Belgium, Sweden, Australia, and Italy also reported several cases of their first monkeypox cases [19]. As of 7 February 2023, there are a significant number of monkeypox infections in nations, including the UK (3735), Spain (7528), Portugal (951), Germany (3692), Brazil (10,758), Canada (1460), Italy (955), France (4128), the USA (29,933), Israel (262), the Netherlands (1260), Argentina (1078), and Australia (144) [4]. Such a high number of cases raises the question as to how monkeypox can survive and transmit in the environment. Given that amoebae are ubiquitous in the environment and we currently lack the means to effectively monitor them or foresee when they might release infectious agents, pathogen-carrying amoebae could pose risks to public health [20].

### 2.1. Acanthamoeba in the Environment

*Acanthamoeba* is known to have an extensive global distribution that spans both aquatic and terrestrial habitats [21,22]. In fact, *Acanthamoeba* is one of the most prevalent protists in the soil. As significant grazers of the bacterial biomass in these settings, these amoebae are thought to regulate not only the variety but also the abundance and turnover of bacterial communities in the soil and plant rhizospheres [23,24,25]. *Acanthamoeba* also releases nutrients locked in the microbial biomass in the soil microbial loop, ultimately promoting plant growth [26].

*Acanthamoeba* can survive in a variety of environments and have been found in swimming pools, bottled water, seawater, ponds, stagnant water, freshwater lakes, saltwater lakes, river water, distilled water bottles, ventilation ducts, the water–air interface, air-conditioning units, sewage, compost, sediments, soil, beaches, vegetables, air, surgical instruments, contact lenses and their cases, the atmosphere (recent decontamination studies), etc., and they have been isolated from the continent of Antarctica [22,27]. Moreover, free-living *Acanthamoeba* spp. have been discovered in a diverse range of animals, including monkeys, dogs, lizards, kangaroos, Indian buffaloes, reptiles, mice etc. [28], and even marine creatures, including fish, amphibians, etc. [29,30,31]. Furthermore, reports of keratitis due to *Acanthamoeba* have been described in animals; however, prospective studies are warranted to comprehend the prevalence of amoebae in animals [32].

### 2.2. Acanthamoeba: The Microbial World’s Trojan Horse

The Trojan horse nature of amoebae, together with their ubiquitous presence in the environment, strengthens our hypothesis. The life cycle of *Acanthamoeba* is comprised of a vegetative trophozoite stage during which amoebae divides mitotically and an inactive dormant cyst stage [22,33,34]. Of note, amoebae cysts are highly impervious to chemicals and physical and radiological conditions and can also be air-borne [13]. This ability of amoebae to phenotypically transform from an active trophozoite form into an air-borne cyst form is of additional concern. *Acanthamoeba* is already known to shelter a wide range of viruses such as poliovirus, mimivirus, enterovirus, coxsackievirus, adenoviruses, and echovirus, amongst others [35], as well as bacteria such as *Coxiella*, *Legionella*, *Mycobacterium*, *Helicobacter*, *Salmonella*, *Pseudomonas*, *Escherichia coli*, *Vibrio*, *Listeria*, *Rickettsia*, *Shigella*, *Pasteurella*, to name a few (reviewed in [11]). Of note, *Acanthamoeba* act as an incubator-type reservoir for microbes, as well as pathogens, where such microbes utilize the amoebae’s defense system to withstand harsh environments and/or elude host defenses and antimicrobial therapy while multiplying within amoebae. Moreover, amoebae are known as a “genetic melting pot”, where the exchange of genes leading to the adaptation of microbes, possibly resulting in greater pathogenicity, may occur [36].

For example, *Acanthamoeba* has also been identified as a yaravirus carrier [37]. The majority of viruses found in free-living amoebae are nucleocytoplasmic large DNA viruses (NCLDV), a group of eukaryotic viruses that also includes the Iridoviridae, Phycodnaviridae, and Asfarvirid families [38]. The majority of viruses associated with *Acanthamoeba* are relatively large, containing 100–1000 genes. In this regard, the genome of the monkeypox virus is around 190 kb and it is an enclosed double-stranded DNA virus [39]. It is interesting to note that orthopoxvirus infection has been associated with numerous reports of antiviral immunity evasion. Numerous genes in the Orthopoxvirus genome code for proteins that interfere with the host cellular communication pathways involved in immunological control and virus recognition [40]. Furthermore, it is significant to note that the monkeypox virus can use a variety of strategies to avoid being targeted and recognized by the host immune system [40,41]. Given that *Acanthamoeba* has been utilized by other pathogens as a training ground (discussed in the following section) and is also often referred to as a “genetic melting pot”, the speculation that the monkeypox virus may be sheltering in these amoebae is highly plausible. The monkeypox virus is a double-stranded DNA virus, it should thus present with a modest mutation frequency [42]. The outbreak strains of monkeypox from 2022 were nonetheless shown to share 46 common mutations. Recent investigations have indicated that the exact mechanisms that cause these mutations are unknown [42]. It is anticipated that additional genomic investigations and evaluations of the experimental evolution of the monkeypox genome and the *Acanthamoeba* genome may reveal information regarding possible genetic exchanges.

Another example of the highly complex interactions of microorganisms comes from a recent report whereby *Morganella morganii* bacteria, isolated from the gut microflora of the Asian water monitor lizard *Varanus salvator* were able to interact, invade, and survive within *Acanthamoeba castellanii*. This study provides another example of the ability of *Acanthamoeba* in acting as a potential vector and aid in the possible transmission of bacteria and/or other microorganisms to their potential hosts, which are found in a variety of places [43]. The specific details of these interactions with various microbes are not fully established, but it is thought that amoebae may be able to facilitate the transmission of pathogenic microbes to prospective hosts and establish infection. Thus, it is probable that monkeypox may be able to survive and multiply inside *Acanthamoeba* with the amoebae acting as a vector and/or reservoir and aiding in its transmission. Additionally, amoebae cysts may also offer resistance to intracellular viruses from the required levels of biocides. If proven, a symbiosis between amoebae and monkeypox may result in significant challenges to the public health and ought to be investigated. Associations between amoebae and the monkeypox virus may also result in transmission to the central nervous system, resulting in more severe cases.

### 2.3. Acanthamoeba as a Training Ground for Pathogens

It is well known that pathogens may have used their capacity to thrive and proliferate inside *Acanthamoeba* as a tool for learning how to avoid the assault of macrophage-mediated death. This is due to the striking similarities observed between how different pathogens such as *Mycobacterium* or *Legionella pneumophila* survive inside human macrophages and *Acanthamoeba*, including the use of similar transcriptional, post-transcriptional, and cellular mechanisms. This suggests that both amoebae and human macrophages may share characteristics that enable the intracellular pathogens to spread infection [35,44].

Interestingly, the natural reservoir of pathogenic *Chlamydiae* is not known [45]. The discovery that *Chlamydophila pneumoniae* can multiply in free-living amoebae in addition to environmental *Chlamydiae* [45] suggests that protozoa may act as an environmental reservoir for *Chlamydiae* in this situation. These studies indicate that *Chlamydiae* may flourish in both amoebae and humans, even though the longevity of this relationship during the amoeba encystment is not yet known [45]. In line with this, it was also shown that the amoeba symbiont *Parachlamydia acanthamoebae* can enter and grow in human macrophages and may therefore have the ability to infect humans [11,46,47].

In addition, *Acanthamoeba* is capable of taking in and housing *Mycobacterium leprae* (known to cause leprosy, a neurological and dermatological disease), and the bacteria can keep both their viability and growth characteristics within the amoebae [48]. Moreover, it has been reported that the quiescent encysted amoebae can shield *M. leprae* from harmful circumstances such as desiccation and high temperature and pH [49]. Similar and prospective studies to determine if the monkeypox virus remains viable and can grow within amoebae trophozoites and cysts are warranted.

### 2.4. The Role of “One Health” in Monkeypox Infection

The need for improved collaboration between the human, animal, and environmental health sectors has been underlined by the apparent inability of global health security to avoid or prepare for the recent COVID-19 pandemic [50]. The monkeypox epidemic is a resurgent viral zoonosis that naturally occurs in highly forested areas in Africa. Monkeypox virus inter-human transmission, albeit rare, is what causes epidemics, particularly in residential and medical settings. The information that is now available, however, points to the possible cessation/reduction of human infections in the absence of recurring zoonotic incursions. Therefore, a key area to focus on in fighting this infection would be to prevent viral transmission from animals to humans [51]. During the 2003 monkeypox outbreak in the US, which was caused after patients came in contact with imported animals (prairie dogs), two patients presented with severe illness [7,52]. One patient had encephalitis that improved during a 14-day hospital stay, and the second had diffused pox lesions, including oropharyngeal lesions that resulted in difficulty in breathing and swallowing [7,52]. The source of this outbreak was a shipment containing nine species of animals, including rodents such as African giant pouched rats, rope squirrels, brush-tailed porcupines, tree squirrels, dormice, and striped mice, some of which were found to be infected with monkeypox. The animals were kept near prairie dogs that were sold as pets before any infection was observed. It is likely that many of these animals interacted with and came across *Acanthamoeba* routinely, given the ubiquitous nature of these amoebae and their omnipresence in the environment [32]. Furthermore, it is plausible that *Acanthamoeba* may be a part of the microbiome of animals, given its presence in diverse environments and the complexity of microbial isolates that have been recovered from amoebae via metagenomic techniques [53]. This is further indicative of the fact that the environment, animals, and humans are all interconnected. Hence, it is necessary to understand that there may be various etiologies, rather than just one etiological agent.

To this end, the use of an amalgamation of approaches may well be the way onward in targeting viruses such as the monkeypox virus that may be using amoebae to shelter and persist in the environments. This notion is further strengthened by the findings from a recent study that investigated different amoebae species that are frequently observed in the environment (namely: *Acanthamoeba* spp., *Dictyostelium discoideum*, *Vermamoeba vermiformis*) as potential reservoirs for the plague causing bacteria reservoir *Yersinia pestis* [54]. Interestingly, these amoebae were isolated from the soil in plague-affected prairie dog burrows. Field-based and laboratory studies were carried out to evaluate the environmental co-occurrence of the study amoebae species with plague epidemics, the prevalence and severity of experimental infections in amoebae, the location of bacteria within amoebae, their viability following phagocytosis, and the replication of bacteria inside trophozoite amoebae [54]. The authors demonstrated the natural phenomena of the co-occurrence of plague-causing bacteria and various amoebae species during an active plague epizootic, suggestive of the complex interactions between bacteria, amoebae, and host immune factors and the environment. Furthermore, amoebae were isolated from prairie dog burrows, which were also the same species that were implicated in monkeypox infection in 2003 [7,52,54].

### 2.5. Monkeypox Virus Interaction with Its Hosts

Monkeypox virus interactions with *Acanthamoeba* and how these interactions impact each symbiont for their prevalence and species dominance are important questions that remain undetermined. The roles of symbionts within their hosts can range from mutually beneficial to parasitic, depending on the endosymbiont and its host. *Acanthamoeba* is considered as the Trojan horse of the microbial world. However, amoebae interactions with smaller microbes is primarily driven by its nutritional needs to ensure that it remains as a vegetatively and metabolically active and multiplying trophozoite. In addition to an increase in its species, this property of *Acanthamoeba* is attributed to the regulation of microbial communities in the environment, in particular for the soil composition/fertility. However, when it feeds on a few selected bacteria/viruses, a selective pressure is exerted that favors phagocytic microbe with mechanisms to resist digestion. Hence, in these cases, amoebae serve as intracellular training grounds for bacteria/viruses to evolve resistance mechanisms against phagocytic killing as well as allowing selected bacteria/viruses to be housed inside *Acanthamoeba* as endosymbionts [11]. Although the impact of *Acanthamoeba*–microbe symbiosis is often described as beneficial to the endosymbionts in terms of enhancing pathogenicity, survival under harsh conditions such as the presence of disinfectants/chemicals as well as resisting physiological/radiological conditions and/or fulfilling their energy needs, the effect of endosymbionts on the host species such as *Acanthamoeba* remains unclear. Among a few studies, it is suggested that the endosymbionts provide benefits to their host *Acanthamoeba* by enhancing *Acanthamoeba* motility and growth that could enhance its environmental prevalence, protecting *Acanthamoeba* from the pathogenic *Legionella* spp. and enhancing *Acanthamoeba* pathogenicity (reviewed in [55]). Using advanced molecular “omics” technologies, there is a need for a holistic approach to understand the microbial community dynamics in natural environments to comprehend effect/function of each symbiont and their prevalence in complex ecological populations.

The pathogenesis of monkeypox virus infection is not well understood, but it is believed to involve a complex interplay between viral and host factors. Monkeypox virus is an enveloped, double-stranded DNA virus that belongs to the genus Orthopoxvirus in the family Poxviridae. Members of the Poxviridae family, such as the monkeypox virus, are thought to exhibit diverse spectra of living and surviving in a host cell [56]. The cellular entry receptor for the monkeypox virus has not been identified, but it is believed to be a member of the laminin-binding integrin family. Once inside the host cell, the virus undergoes a series of replication steps, including the expression of early and late viral genes, DNA replication, and the assembly of virions [57]. The virus replicates in the cytoplasm of infected cells and produces two distinct forms of infectious particles, intracellular mature virions and extracellular enveloped virions [58]. The immunohistochemical and histopathological tests found that the monkeypox virus antigens were identified in ovarian, brain, heart, kidney, liver, pancreatic, and lung tissues [59], suggesting extensive tissue infection and damage. The host immune response to monkeypox virus infection is complex and involves both innate and adaptive immune mechanisms. Innate immune cells, such as dendritic cells, macrophages, and natural killer cells, are activated early in the infection and secrete pro-inflammatory cytokines and chemokines, which recruit additional immune cells to the site of infection. The adaptive immune response to the monkeypox virus involves the production of virus-specific antibodies and T cells, which can recognize and eliminate infected cells [60]. The Poxviridae family virus develops many strategies to escape the host’s immune response to infection. Natural killer (NK) cells are supposed to kill virus-infected cells by secreting cytokines that would stimulate the activity of other cell types, such as T cells and dendritic cells [61]. Monkeypox virus infection can induce NK cell changes such as an increment in the number of all NK subsets in non-human primates [62]. Moreover, following the monkeypox virus infection a delayed or reduced expression of chemokine receptors on each NK cell subset suggested its immune evasion response [40]. It was also reported that the monkeypox virus has a safe avoidance component and the avoidance process utilized by the monkeypox virus ensures the viral store is resistant by repressing the activation of CD4+ and CD8+ T cells after interaction with monkeypox-virus-infected cells [63].

Although the precise mechanisms of monkeypox evasion of *Acanthamoeba* phagocytic killing require experimental investigations, based on genome analysis and earlier investigation using human cells, several viral proteins have been found to be essential for entry, release, and host cell modulation. In this regard, several cell modulatory proteins have been identified that may also play a role in monkeypox evasion of *Acanthamoeba* intracellular killing. These include: J3R (chemokine binding protein), J2L (cytokine response-modifying protein B), D9L (ankyrin repeat domain containing protein), CP77 (type I interferon (IFN) evasion protein), F3L (RNA-binding protein E3), H1L (dual specificity protein phosphatase H1), D3R (EGFR binding protein), D11L (Protein C6), C7L (Protein F1), B16R (soluble IFN-alpha receptor), C1L (IFN antagonist K1L), B13R (protein B13), B9R (soluble IFN-γ receptor B8), P1L (protein N1), C6R (protein K7), A37R (MHC modulating protein), A41L (protein A41 (chemokine binding protein), and A47R (TLR inactivating protein); however, studies are needed to determine the role of the aforementioned proteins in evading phagocytic killing of *Acanthamoeba* [64].

As in the case of other microorganisms, amoebae have been recognized as biological “Trojan horses” for viruses and have been charged with increasing virulence and protecting human viruses against environmental harshness. Amoeba-infecting viruses are not uncommon, and several have been isolated from amoeba cultures, such as adenoviruses [65] and enteroviruses [66]. Additionally, previously we reported that the presence of heat shock proteins in *Acanthamoeba* might be allowing long-term survival and long-distance transmission of the SARS-CoV-2 virus [67]. In any case, the receptors and the internalization pathways for amoeba–monkey virus interaction have not been described. However, recent genomic analysis of *Acanthamoeba* showed the presence of laminin-binding protein as one of the major parasites’ adhesins [68]. Given the fact that the entry of the monkeypox virus into host cells is mediated by the viral envelope glycoproteins, we suggest this could interact with laminin-binding proteins on the surface of *Acanthamoeba* allowing its entry and facilitating its transmission. Notably, monkeypox virus has a relatively large genome of about 196,858 base pairs, encoding 190 open reading frames needed for viral replication. It is logical that viral replication can only take place during the metabolically-active trophozoite stage of the *Acanthamoeba* as the virus requires the functional host cell machinery to synthesize its DNA, early and later proteins, virion assembly, trafficking, etc. The cyst stage is inactive or exhibit minimal metabolic activity. For example, viral entry into cells is likely dependent on phagocytic uptake (absent in the cyst stage) and involves actin. Once inside, viral proteins and enzymes promote synthesis of early proteins, DNA replication, transcription factors, late genes, structural proteins, and enzymes and virion genomes are processed and assembled into nascent virions that contain all enzymes, factors, and genetic information needed for a new infectious cycle. Hence, it is likely that, once infected with monkeypox, the metabolism of *Acanthamoeba* and monkeypox virus are functionally intertwined; however, future experimental studies are needed to determine this.

## 3. Future Perspectives and Conclusions

As amoebae can harbor different microorganisms and/or pathogens (however, whether amoebae can harbor monkeypox needs to be determined), these pathogens may spread across our ecosystem and cause infections in humans and animals, leading to highlighting the concept of “one health,” in which the wellbeing of humans, animals, and the environment is intertwined. Numerous studies of various metabolic, proliferative, and survival systems in eukaryotic species have been elucidated through the utilization of *Acanthamoeba* as a model [69]. Furthermore, these amoebae are unique as they exhibit invasive strategies and capture their target by phagocytosis as well as have the ability to switch their phenotypes according to diverse environmental conditions. Studies to understand if viruses such as the monkeypox virus can survive inside *Acanthamoeba* are necessary and will pave the way for the development of disinfectants that may be effective against both the viral agent or agent of interest and the host, *Acanthamoeba*. A complete understanding of the molecular underpinnings of these interactions, such as variations in the gene expressions or receptors used by microorganisms, may also help to promote a one-health approach and shed light on potential ways to manage pathogenicity and drug resistance. Given this, we suggest that amoebae cysts should be targeted as well to offer robust infection control approaches. The concept of utilizing antiamoebic approaches in containing viruses such as monkeypox should be seriously considered. Nonetheless, prospective research is needed to understand the efficacy of targeting amoebae with disinfectants, as is investigating the interaction of monkeypox with amoebae.

## Data Availability

Data sharing is not applicable to this article.
